# Cannonball Pulmonary Opacities Disclosing a Granulomatosis With Polyangiitis (GPA) With C-Antimyeloperoxidase (C-Anti-MPO) Antineutrophil Cytoplasm Antibodies (ANCAs)

**DOI:** 10.7759/cureus.25281

**Published:** 2022-05-24

**Authors:** Meriem Rhazari, Hiba Ramdani, Sara Gartini, Othman Moueqqit, Gokul Paidi, Mohammed Musallam, Afaf Thouil, Hatim Kouismi

**Affiliations:** 1 Pulmonology, Mohammed VI University Hospital, Oujda, MAR; 2 Medicine, Mohammed VI University Hospital, Oujda, MAR; 3 Faculty of Medicine and Pharmacy, Mohammed First University, Oujda, MAR; 4 General Medicine, Faculty of Medicine and Pharmacy, Mohammed First University, Oujda, MAR; 5 Family Medicine, The Medina Clinic, Grandview, USA; 6 Pneumology, Centre Hospitalier Universitaire (CHU) Mohammed VI, Oujda, MAR; 7 Faculty of Medicine, Mohammed First University, Oujda, MAR; 8 Respiratory and Allergic Diseases, Mohammed VI University Hospital, Oujda, MAR

**Keywords:** cannonball, mpo-anca, anca vasculitis, granulomatosis with polyangiitis, rounded pulmonary opacities

## Abstract

Granulomatosis with polyangiitis (GPA) is a necrotizing granulomatous vasculitis of medium- and small-caliber vessels associated with the presence of antineutrophil cytoplasm antibodies (ANCAs) and antibodies specific for proteinase 3 (anti-PR3). The interest of this case lies on the fact that these antibodies are directed against myeloperoxidase revealed by the presence of scattered multiple pulmonary nodules.

We report a 65-year-old-female patient who presented with a productive cough with mucus sputum associated with a cephalea for six months. The chest x-ray showed multiple pulmonary nodules, first suggesting a neoplastic origin. The initial etiological assessment was non-contributory. A month later, the patient developed pulmonary condensations and ocular signs. The etiological assessment then found ANCA anti-myeloperoxidase (anti-MPO)-GPA. A good knowledge of the clinical and radiological signs of GPA is important to quickly guide the diagnosis that will condition the prognosis of this disease.

## Introduction

Multiple pulmonary nodule images usually raise the suspicion of metastases of a distant primary tumor, but several inflammatory and infectious conditions that may lie behind these radiological appearances should be excluded as well [1]. Granulomatosis with polyangiitis (GPA) is one of these conditions that should be considered facing these features [1].

GPA, formerly known as Wegener's disease, is a necrotizing vasculitis combining inflammation of the medium- and small-caliber vessel walls and peri- and extravascular granulomatosis [2]. Immunologically, antineutrophil cytoplasm antibodies (ANCAs) against the proteinase 3 (anti-PR3) are the most specific to the disease [2]. Clinically, in its complete form, otolaryngologic and pneumorenal syndromes [3] characterize granulomatosis with GPA. The diagnosis is based on a set of clinical, histological, and biological arguments [4]. We report a case of GPA revealed by multiple scattered pulmonary nodules with antimyeloperoxidase (anti-MPO)-specific c-ANCAs.

## Case presentation

**Figure 1:**
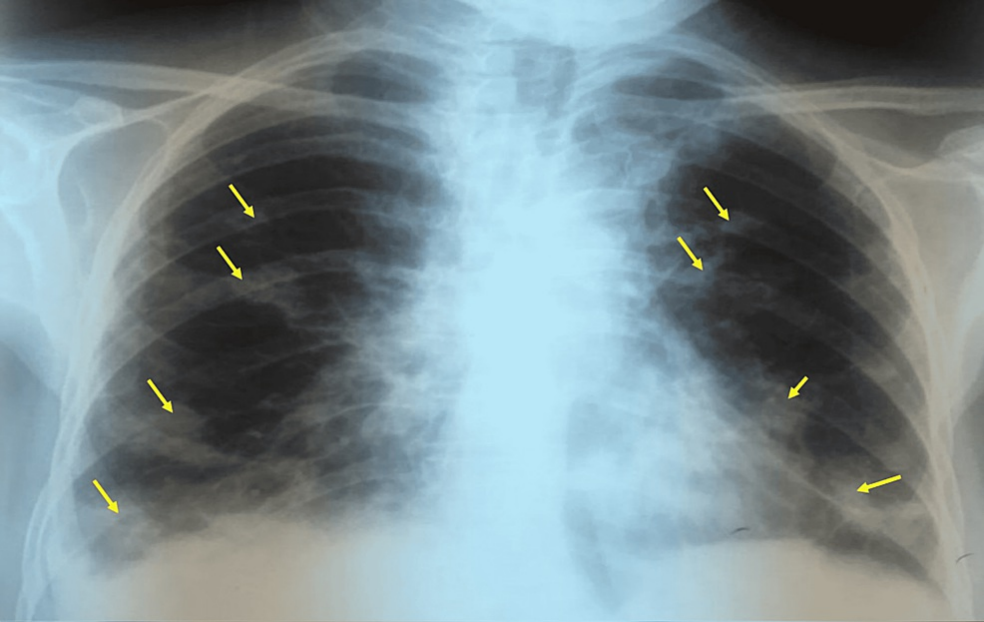
Anteroposterior chest x-ray (CXR) evaluating the cause for chronic productive cough showing multiple and disseminated rounded macronodular opacities of different sizes

A 65-year-old female patient, previously treated for pulmonary tuberculosis 30 years ago, presented with chronic cough producing white to yellowish mucous sputum associated with a frontal headache of variable intensity. The patient also had a history of asthenia and non-measured fever but declined any other symptoms such as wheezing/shortness of breath and no use of medications. Upon admission, the patient deteriorated with a World Health Organization (WHO) score of 3, with the following vital parameters: systolic blood pressure of 130 mmHg, diastolic blood pressure of 70 mmHg, heart rate of 90 beats/min, and arterial oxygen saturation of 94%. The clinical examination of the pleuropulmonary, lymph node, cardiac, neurological, cervical, ophthalmological, cutaneous, and gynecological systems was normal. Chest x-ray (CXR) (Figure 1) demonstrated diffused rounded parenchymal opacities of different sizes.

**Figure 2:**
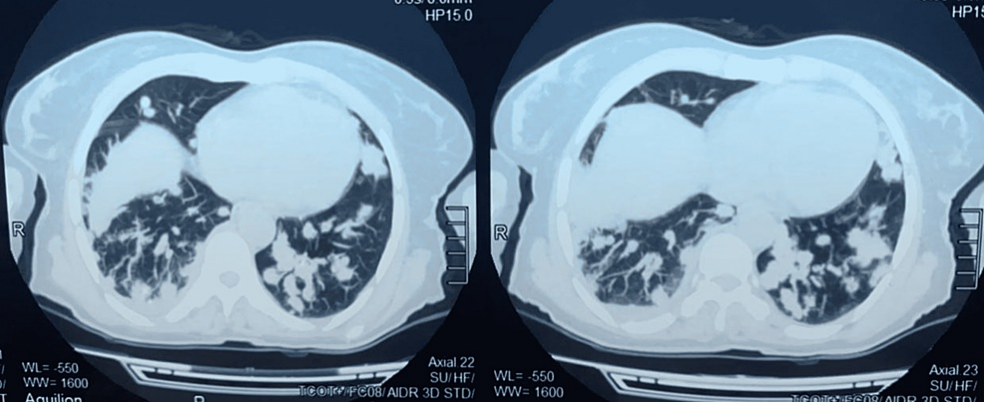
Thoracic CT scan, parenchymal window: multiple rounded lesions of different sizes disseminated to the two pulmonary hemifields

A thoraco-abdominopelvic CT scan was performed in search of a primary cause, which demonstrated the presence of multiple bilateral intraparenchymal nodules with soft tissue density - some with spiculated borders and no visible primary at the thoracic, abdominal, and pelvic levels (Figure 2).

Several diagnostic hypotheses can be considered here, which include pulmonary metastases, a disease of an infectious origin such as tuberculosis, staphylococcus infection, or a systemic disease such as Wegener's arthritis and rheumatoid arthritis. In this case, the neoplastic origin was the most likely one considering the age, the deterioration of the general condition, and the presence of weight loss. Likewise, the diagnosis of pulmonary tuberculosis cannot be ruled out, especially in endemic areas.

**Figure 3:**
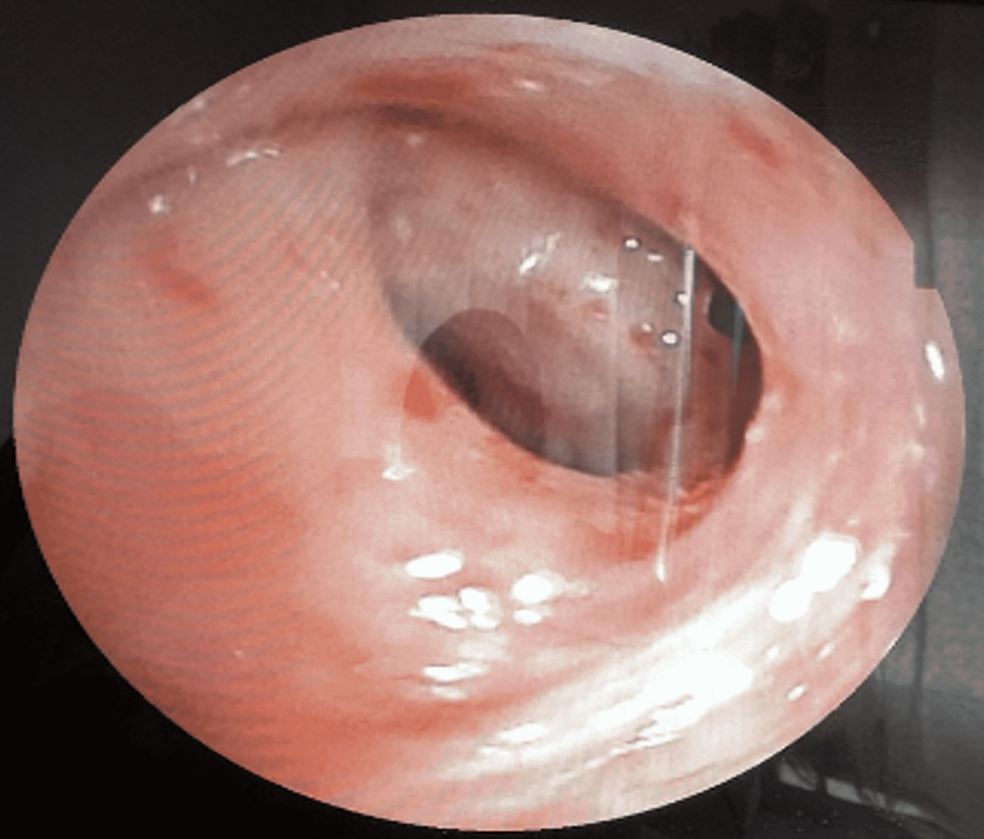
Bronchoscopic appearance shows the thickening of the interculminolingular spur with extrinsic compression at the culmen level

A laboratory workup showed that the C-reactive protein (CRP) was elevated to 114 mg/L (<5 mg/L). Neutrophils were increased to 18,000 elements/mm^3^, and eosinophils were normal. Viral serologies (HIV, venereal disease research laboratory/*Treponema pallidum* hemagglutination [VDRL/TPHA], and hepatitis B and C), tumor markers (CA125, CA199, CA153, and angiotensin-converting enzyme [ACE]), and interferon-gamma release assay (IGRA) were normal. Microbiological analysis of the sputum is sterile. Sputum and bronchial aspiration fluid were negative for Koch's bacilli. The bronchoscopy showed a thickening of the interculminolingular spur with extrinsic compression at the culmen level, which makes its orifice non-catheterizable (Figure 3). The analysis of fibroscopic samples (bronchial aspirations, bronchial biopsies, and search for mycobacteria) shows only an inflammatory aspect.

**Figure 4:**
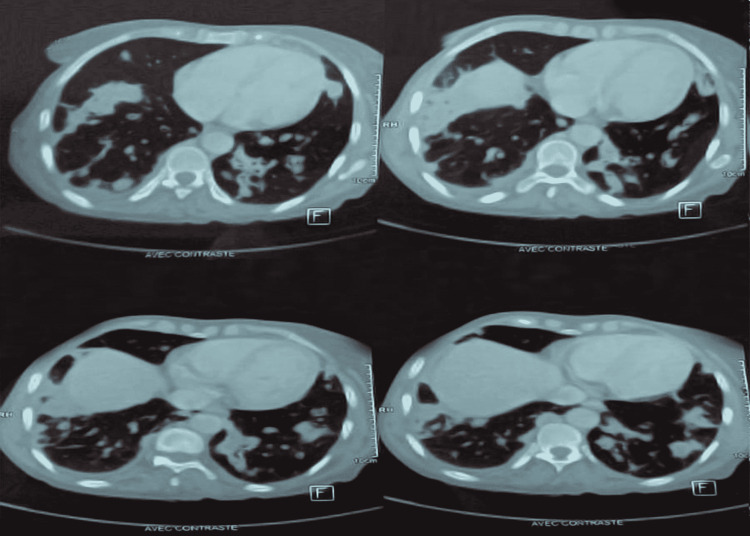
Chest computed tomography, parenchymal window: multiple foci of bilateral parenchymal condensation

**Figure 5:**
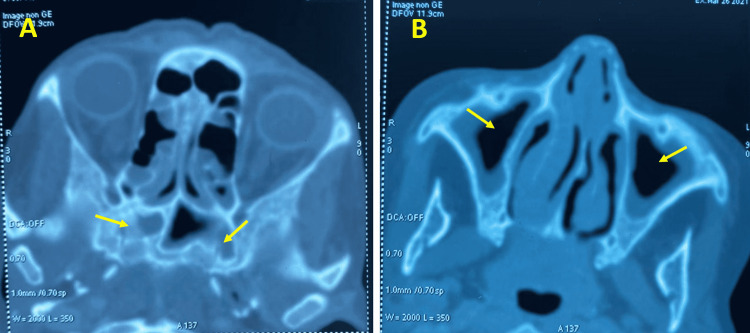
Axial sinus scan. (A) Filling of the sphenoid sinus. (B) Frame thickening of the maxillary sinuses.

The patient had received a therapy of broad-spectrum antibiotics (Amoxicilline and clavulanic acid + macrolides) for 10 days but without clinical improvement. Following the lack of diagnostic orientation at the end of these first-line explorations, it was decided to carry out a positron emission tomography (PET) scan to characterize the evolution of the pulmonary lesions and the search for a primary neoplastic localization. However, as it was not available, a cerebral and cervico-thoraco-abdominopelvic CT scan was performed one month after the initial scan. The evolution of the radiological images (one month) was characterized by the confluence of the nodules into a focus of bilateral pulmonary parenchymal condensation (Figure 4) with the presence of a filling of the sphenoid sinuses and a thickening in the two maxillary sinuses and the left sphenoid sinus (Figure 5). In light of this, the suggested diagnosis included infectious, auto-immune, hematologic, or neoplastic origins.

**Figure 6:**
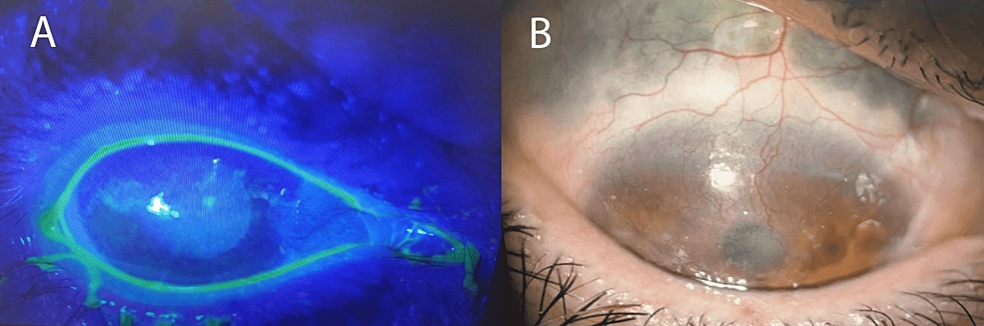
(A) Infected keratitis. (B) Superior subconjunctival scleromalacia with corneal neovascularization.

In addition, the calcium and phosphate levels and the conversion enzyme were normal; the electrocardiogram (EKG) and the transthoracic echography were normal; the rest of the laboratory workup showed no abnormalities, while the biopsy of the accessory salivary gland showed a dry syndrome. A specialized ENT (ear, nose, and throat) examination showed the presence of pus in the middle meatus with hypoacusis, and the ophthalmological examination, which initially appeared normal, showed an aspect of sequential corneal neovascularization on the left one month later, an infected keratitis associated with scleromalacia on a likely scleritis (Figure 6).

**Figure 7:**
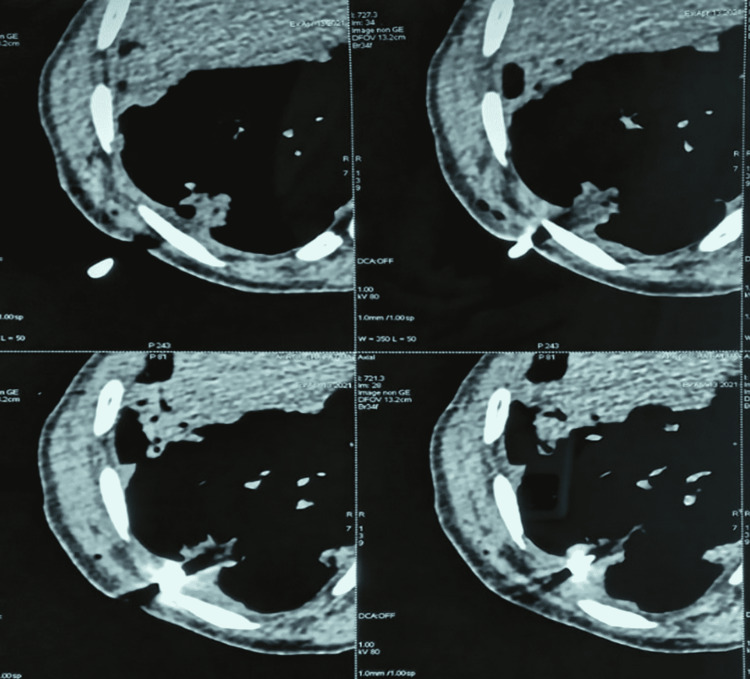
Chest CT scan, mediastinal window: the site of the scan-guided lung biopsy

The immunological assessment showed abnormal levels of the cytoplasmic antineutrophilic antibody (c-ANCA) with a significant increase in the antimyeloperoxidase antibodies (anti-MPO). The cytobacteriological examination of urine (CBEU) revealed microscopic hematuria without leukocyturia or proteinuria. The respiratory function tests did not show any obstructive or restrictive syndrome. An MRI of the spinal cord was performed along with electromyography (EMG), but both revealed no abnormalities. Furthermore, to exclude an association with malignant pathology and to confirm the vascular nature of lung involvement, a lung biopsy under CT scan in the center of a nodule in the right lower lobe was performed (Figure 7).

**Figure 8:**
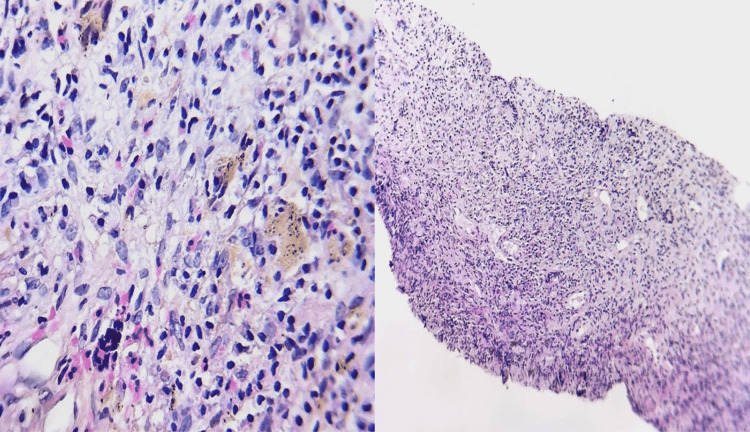
Epithelioid granuloma without caseous necrosis with a few giant cells in vascular contact

Histopathological examination of the biopsy sample revealed an epithelioid granuloma without caseous necrosis with a few giant cells in vascular contact (Figure 8). The Ziehl-Neelsen and periodic acid-Schiff (PAS) stains were negative, and the bacteriological study of the specimen including the search for Koch’s bacilli was also negative.

The diagnosis of GPA was made according to the American College of Rheumatology (ACR) criteria in view of the pulmonary, otolaryngological, ophthalmological, and renal manifestations associated with the positivity of the c-ANCA anti-MPO and the presence of granuloma on the pulmonary biopsy. The patient was treated with a methylprednisolone bolus (500 mg/day for three days) followed by oral corticosteroid therapy (1 mg/kg) and immunosuppressive treatment. The clinical evolution was marked by the regression of general signs and the normalization of the CRP levels within one month.

## Discussion

According to Chapel Hill's revised criteria in 2012, GPA is defined as a necrotizing granulomatous inflammation of the upper and lower airways with necrotizing of small- and medium-vessel vasculitis [1]. In 1936, a German pathologist, Friedrich Wegener, described the clinical triad associated with this disease [4]. Cytoplasmic antineutrophil antibodies (ANCA) were identified in 1980 [5]. The disease was then known as Wegener's granulomatosis. Since 2011, the use of descriptive and etiological nomenclature is recommended by the ACR, thus Wegener's granulomatosis is called granulomatosis with polyangiitis [6].

GPA is a rare condition with a prevalence ranging between 1/42000 and 1/6400 people worldwide and an annual incidence of two to 12 cases per million [7]. It affects both genders, and the average age at onset is 45 years. Some forms have been described among the elderly and children [2]. The precise etiology is unknown, and environmental factors such as dust inhalation or exposure to silica are most likely involved but are seen in only 10% of patients with GPA [5]. Infectious agents may be involved in the triggering of the disease in genetically predisposed individuals [5]. Different immunological abnormalities have been identified in recent years, involving the production of ANCA against PR3 in about 80% of patients with GPA and against MPO in about 10% of patients with GPA [8].

A distinction is made between the so-called diffuse systemic forms, in which vascular phenomenon is characterized by life-threatening manifestations and localized forms in which granulomatous inflammation is dominant [9]. The transition from a localized/limited form to a generalized/diffuse form and vice versa is possible during the evolution of the disease [2]. Constitutional signs (fever, asthenia, and weight loss) are common (50%) but nonspecific [6]. Otorhinolaryngologic (ENT) signs reveal GPA (70%-100%) but are often unrecognized early in the disease. These may include crusted rhinorrhea, sinusitis, chronic otitis media, or facial cartilage involvement with deformities causing a saddle nose [5].

Pulmonary involvement occurs in 50%-90% of patients. It is not very specific, with a cough (75% of cases), sometimes with purulent sputum, moderate dyspnea (50%), and hemoptysis (30%-40%) indicating a focal lesion (excavated nodule) or diffuse alveolar hemorrhage [10]. Chest pain is usually related to pleural origin (exudative pleurisy, pneumothorax complicating an excavated nodular parenchymal lesion, or bronchopleural fistula) [11]. Radiologically, nearly 50% of patients present bilateral or unilateral pulmonary infiltrates. Sometimes, large intraparenchymal nodules progressively increase in number and size and tend to excavate. Pleural effusion has also been reported in 15%-20% of cases [9,12] and more rarely intra-alveolar hemorrhage [13]. Its signs are common to several pulmonary diseases such as pulmonary tuberculosis, sarcoidosis, fungal infections, and pulmonary metastasis, all of which should be excluded before the GPA diagnosis is made [14].

In our context, Morocco is an endemic country for tuberculosis; so, it is crucial to diagnose GPA since the therapeutic approach is different between the two diseases. Moreover, the therapeutic choice consisting of immunosuppression aimed at GPA can aggravate an underlying TB. Renal GPA is observed in 40%-100% of cases. It is a major prognostic factor that determines both the renal functional prognosis and the vital potential of the disease. It is a pauci-immune necrotizing and crescentic glomerulonephritis that can be suspected clinically if the patient presents with hematuria, proteinuria, cellular casts on urine cytology, and renal failure [15].

Ophthalmic involvement occurs in 30%-50% of patients with polyangiitis granulomatosis and can involve any part of the eye from simple conjunctivitis to necrotizing scleritis, corneal ulcerations, or diplopia. The disease may also manifest as uveitis or optic neuritis [16]. Other systemic involvement may develop, such as involvement of the nervous, articular, and mucocutaneous systems, but these are less common.

Laboratory evaluation reveals a constant increased C-reactive protein along with other inflammatory markers [11]. The presence of PR3-specific cytoplasmic ANCA is a major diagnostic argument, and their sensitivity is higher (85%-90%) in generalized and active forms of the disease and lower in localized forms (~60%) and remission (~40%) [11]. Specific anti-MPO ANCAs are uncommon in Wegener's disease [17]; so, the differential diagnosis is microscopic polyangiitis. Some clinical and biological findings are suggestive of either disease, but biopsy has an essential diagnostic role.

Chen et al. found a higher prevalence of females and a higher proportion of systemic involvement among the patients with Wegener's disease with positive anti-MPO [18]. The absence of ANCA, however, does not exclude the diagnosis, which must then be supported by sufficient histopathological and/or clinical arguments. Histological study of a lung, kidney, or nasal biopsy may reveal three criteria, all of which may rarely be found together: histiocytic granuloma aligned in a palisade, vasculitis of medium- and small-caliber vessels, and necrosis of the inflammatory tissue [13]. The diagnosis of granulomatosis with polyangiitis is then based on the criteria established by the ACR (Table 1) [14,19-21].

**Table 1:** ACR/EULAR 2017 Provisional Classification Criteria for GPA ACR: American College of Rheumatology; EULAR: European League Against Rheumatism; GPA: Granulomatosis with polyangiitis; C-ANCA: Cytoplasmic antineutrophil cytoplasmic antibody; PR3-ANCA: Proteinase 3-ANCA. Source: Ref. [22].

Items	Score
Bloody nasal discharge, crusting, ulcers, or sinonasal congestion	3
Nasal polyps	-4
Cartilaginous involvement	2
Reduction in hearing or hearing loss	1
Red or painful eyes	1
C-ANCA or PR3-ANCA	5
Eosinophil count ≥1 (x 10^9^/L)	-3
Chest imaging showing nodule, mass, or cavitation	2
Granuloma on biopsy	3
The ACR/EULAR 217 Provisional Classification Criteria for GPA	Sum ≥ 5

The score in our case is 13 (chronic sinusitis - 1, hearing loss - 1, ocular involvement - 1, C-ANCA - 5, nodules on thoracic imaging - 2, and granuloma on biopsy - 3). The initial treatment to achieve remission was combining corticosteroid therapy and immunosuppressive therapy (such as cyclophosphamide or rituximab). Maintenance therapy is based on azathioprine or methotrexate with a relapse rate within five years of initial remission up to 50%.

## Conclusions

The presentation of this case reminds us that the radiological appearance of multiple scattered pulmonary nodules is not always of neoplastic origin. GPA is a potentially fatal disease with a low survival rate of only 20% if left untreated. Early detection of the disease improves the prognosis.
